# Organ-Dysfunction Markers in Mild-to-Moderate COVID-19 Convalescents

**DOI:** 10.3390/jcm13082241

**Published:** 2024-04-12

**Authors:** Aleksandra Wiśniewska, Aleksandra Kijak, Karolina Nowak, Michalina Lulek, Agata Skwarek, Milena Małecka-Giełdowska, Marcin Śmiarowski, Szczepan Wąsik, Olga Ciepiela

**Affiliations:** 1 Students Scientific Group of Laboratory Medicine, Medical University of Warsaw, 02-097 Warsaw, Polandmichalina.lulek@uckwum.pl (M.L.); agata.skwarek1@gmail.com (A.S.); marcin-smiarowski1998@wp.pl (M.Ś.); s085980@student.wum.edu.pl (S.W.); 2 Clinical Laboratory of Central Teaching Hospital, University Clinical Center of Medical University of Warsaw, 02-097 Warsaw, Poland; milena.malecka@wum.edu.pl; 3 Department of Laboratory Medicine, Medical University of Warsaw, 02-097 Warsaw, Poland

**Keywords:** COVID-19, long COVID, lung fibrosis, neurodegeneration, renal function

## Abstract

**Background**: A coronavirus disease 2019 (COVID-19) outbreak led to a worldwide pandemic. COVID-19 not only caused acute symptoms during the severe phase of the disease, but also induced long-term side effects on the functioning of many organs and systems. Symptoms that were associated with the disease and present at least 3 months after recovery were named long COVID. The aim of this study was to assess if mild-to-moderate COVID-19 may lead to the dysfunction of respiratory, cardiovascular, neural, and renal systems in healthy blood donors who recovered from the disease at least 6 months earlier. **Methods:** Here, we examined 294 adults among volunteer blood donors divided into convalescents (*n* = 215) and healthy controls (*n* = 79). Concentrations of soluble CD163, TGF beta, Lp-PLA2, NCAM-1, S100, NGAL, and creatinine were measured either by ELISA or automated methods. The probability value *p* < 0.05 was considered as statistically significant. **Results:** We found significant differences in Lp-PLA2, S100, and NCAM-1 between convalescents and never-infected subjects. Lp-PLA2 and NCAM-1 were lower, and S100 higher, in convalescents than in the control group. **Conclusion:** Mild-to-moderate COVID-19 convalescents are at a low risk of developing lung fibrosis or chronic kidney disease. However, they should regularly carry out their prophylaxis examinations for early detection of possible negative outcomes of COVID-19.

## 1. Introduction

Coronavirus disease 2019 (COVID-19) is a disease caused by the severe acute respiratory syndrome coronavirus 2 (SARS-CoV-2). The outbreak of the disease, which led to a worldwide pandemic, began in China at the end of 2019. In consequence, the disease affected hundreds of millions of people, leading to the death of over 80 million individuals and exerting a significant influence on public health. COVID-19 was initially associated mainly with severe upper respiratory tract infection, but turned out to affect many of the human organs and systems, including nervous, cardiovascular, and renal systems. A large number of COVID-19 survivors have presented with different disease syndromes for a long time after recovery from the acute viral infection. As these convalescents become a serious burden on the health care system, the prolonged health problems associated with previous SARS-CoV-2 infection have been classified as a syndrome named long COVID [1,2]. Data in the literature suggest that the prevalence of long COVID is between 9% and 63% of recovered subjects, depending on the source of data and syndrome definition [3].

One of the most often-described complications of COVID-19 is pulmonary fibrosis. It appears when excessive deposition of the extracellular matrix can be found within the interstitium of the lungs. Lung fibrosis during the course of COVID-19 is directly associated with either the negative impact of the virus on alveolar epithelial cells or the promotion of the inflammatory response and induction of macrophages and monocytes, which release several inflammatory mediators like tumor growth factor beta (TGFβ), platelet-derived growth factor (PDGF), and soluble CD163 (sCD163). Excessive proliferation of fibroblasts and the transition of epithelial cells into mesenchymal cells are also observed in the lung tissue, which together leads to fibrosis and loss of respiratory function [4,5]. Despite significant restoration of pulmonary function in COVID-19 convalescents, markers of lung fibrosis may be detectable at least 6 months after recovery [4,6].

The negative impact of SARS-CoV-2 on endothelial cells is not limited to the lungs. Acute COVID-19 was recognized as a multi-system dysfunction, with a severe influence on the endothelium that may lead to the development of autoimmunity, activation of disseminated coagulation, dysregulation of the renin–angiotensin–aldosterone system, or even sepsis [7]. Increased expression of several cardiovascular risks like syndecan-1, endothelin-1, soluble P-selectin, soluble platelet endothelial cell adhesion molecule 1 (sPECAM-1), vascular endothelial growth factor (VEGF), vascular endothelial (VE)-cadherin, von Willebrand factor (vWf), tissue factor (TF), soluble (s)E-selectin, and others have been found to be associated with SARS-CoV-2 infection and long COVID [8]. One factor, lipoprotein-associated phospholipase A2 (Lp-PLA2), is a well-known cardiovascular risk marker that binds to low-density lipoprotein (LDL), lipoprotein (a), and very-low-density lipoprotein (VLDL) [9]. Moreover, Lp-PLA2 plasma concentrations appear to be useful as a risk stratification marker in the course of COVID-19 [10].

Another system which SARS-CoV-2 has been found to affect is the nervous system. Data in the literature indicate that cognitive dysfunction appeared in at least 25% of patients with COVID-19, and other neural dysfunctions have been recognized in over 75% of COVID-19 survivors 3 months after recovery. Cognitive impairment, mainly memory disorders, fatigue, or attention disorders, persisted for at least 2 years after recovery, and is one of the characteristic features of long COVID. The connection between the nervous system and SARS-CoV-2 infection includes inflammation of the nervous system, induction of autoimmune brain disease, blood–brain barrier disruption, and, rarely, neuroinvasive infection [11,12,13].

Endothelial injury in the course of COVID-19 affects the kidneys as well [8]. The risk of kidney failure in survivors increases with the severity of COVID-19. Both acute kidney injury (AKI) and chronic kidney disease (CKD) have been associated with SARS-CoV-2 infection. Interestingly, in a study that included almost 90,000 participants, a decreased glomerular filtration rate was detected six months after recovery in over 13% of patients [14]. The risk of developing CKD is higher in patients who developed AKI during the active phase of COVID-19 infection. The markers that are used to assess the glomerular filtration rate and overall function of the kidneys are creatinine and neutrophil gelatinase-associated lipocalin (NGAL) [15]. Development of AKI in the course of COVID-19 has been confirmed in many studies; however, researchers argue that serum creatinine and NGAL measurements are as useful as urine measurements of these markers [16,17].

As COVID-19 is a disease that impacts different organs and systems, it is important to examine its long-term effects on the overall health of affected individuals. Thus, the aim of this study was to assess if mild-to-moderate COVID-19 leads to dysfunction of the respiratory, cardiovascular, nervous, and renal systems of healthy blood donors who had recovered from the disease at least 6 months earlier.

## 2. Materials and Methods

### 2.1. Control Group and Study Group

There were 294 adult participants recruited to this study from volunteer blood donors in Warsaw’s Blood Centre from August 2021 to April 2022. All of them were examined by a physician and qualified as healthy and able to donate blood in accordance with the guidelines of the Polish Minister of Health (Table 1). Additionally, they were all tested for SARS-CoV-2 infection and received a negative PCR result at the day of admission. Among all enrolled subjects, 147 of them declared previous mild-to-moderate SARS-CoV-2 infection at least 6 months before blood donation (minimal period of time required between the disease and blood donation, which was confirmed by a positive PCR test result). The mild disease was defined as a lack of symptoms of lower respiratory disease (shortness of breath (dyspnoea) and abnormal chest imaging) and an oxygen saturation measured by pulse oximetry (SpO2) ≥ 94%. The moderate COVID-19 was defined as symptoms of lower respiratory disease with SpO2 ≥ 94%. The 147 individuals declared no SARS-CoV-2 infection. In the next step, all the participants were tested for antibodies against the SARS-CoV-2 N protein in their blood. Based on the anti-N SARS-CoV-2 antibodies evaluation, among the 147 subjects who declared no contact with SARS-CoV-2 and no signs of respiratory tract infection, 68 had a positive result of antibodies and were eventually classified as asymptomatic convalescents. Thus, the study group (convalescents) consisted of 215 subjects, and the control group of 79 subjects. All the participants were at the age of 18–65 years old, and all their specific data, including age and sex, as well as characteristics (body weight, blood pressure, etc.), were not provided.

### 2.2. Methods

A serum sample was obtained from every participant at the day of admission to this study and stored at −70 °C until analysis. Each sample was tested and the concentrations of soluble CD163, TGF beta, Lp-PLA2, NCAM-1, S100, NGAL, and creatinine were measured. The concentrations of molecules (soluble CD163, TGF beta, Lp-PLA2, and NCAM-1) were measured with enzyme-linked immunosorbent assays (Proteintech (Planegg-Martinsried, Germany) or the BOSTER PicoKineTM (Pleasanton, CA, USA) ELISA kit or catalogue numbers sCD163 KE00190, TGF beta KE00002, NCAM-1 EK0706, and Lp-PLA2 EK1637). The S100 protein concentration was measured with electrochemiluminescence immunoassay (ECLIA) on the Cobas e 801 (Roche Diagnostics, Basel, Switzerland); NGAL and creatinine concentrations were assessed using Cobas c503 (Roche Diagnostics). For the detection and titer measurement of anti-SARS-CoV-2 N protein antibodies, expressed as COI, the electrochemiluminescence immunoassay (ECLIA) on the Cobas e 801 (Roche Diagnostics) was used.

### 2.3. Statistical Analysis

The statistical analysis was performed with GraphPad Prism 9 software. The results of all parameters had a non-normal distribution according to the Shapiro–Wilk, Anderson–Darling, Kolmogorov–Smirnov–Lillefors, and D’Agostino–Pearson tests. The Mann–Whitney U test was used for the statistical analysis of the results. The results of the tested molecules measurements are expressed as the median (M), first quartile (Q1), and third quartile (Q3). The probability value *p* < 0.05 was considered as statistically significant.

### 2.4. Bioethical Committee Opinion

The study protocol was accepted by the Bioethical Committee of the Medical University of Warsaw (protocol code AKBE/136/2021, date of approval 6 September 2021).

## 3. Results

### 3.1. Markers of Lung Fibrosis and Pulmonary Macrophages Activation

**Figure 1:**
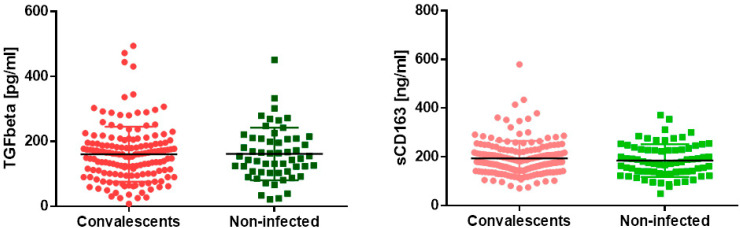
TGF-β and soluble CD163 concentrations in sera of COVID-19 convalescents and non-infected subjects. Differences were not statistically significant, *p* > 0.05.

As a marker of lung fibrosis, the concentration of TGFβ was measured. We found no difference in the marker concentration between convalescents and non-infected subjects. A similar result was obtained when analyzing soluble CD163 concentrations as a marker of pulmonary macrophages activation (Figure 1). Exact values of all measurements are summarized in Table 1.

### 3.2. Vascular Dysfunction Marker

**Figure 2:**
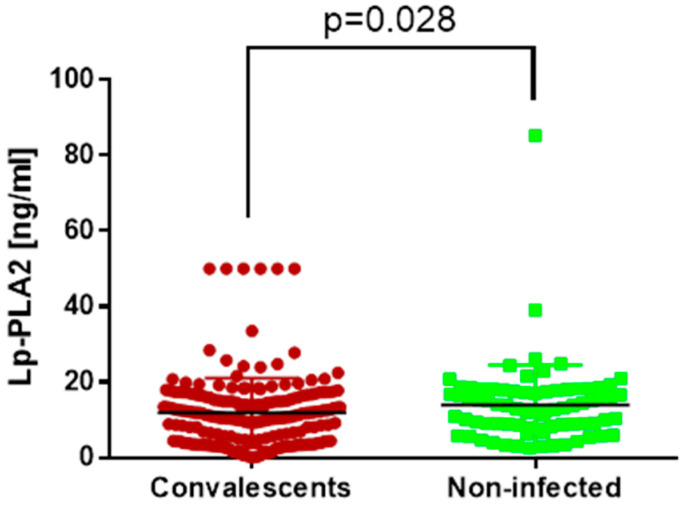
Lp-PLA2 concentrations in sera of COVID-19 convalescents and non-infected subjects. Differences are statistically significant, *p* = 0.028.

Lp-PLA2 in our study served as a marker of vascular function. We found that convalescents had a significantly lower concentration of Lp-PLA2 than subjects who have never suffered from SARS-CoV-2 infection (Figure 2). Exact values of the measurements are summarized in Table 1.

### 3.3. Nervous System Markers

**Figure 3:**
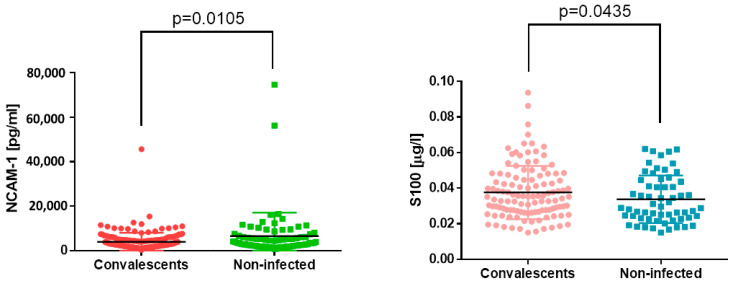
NCAM-1 and s100 concentrations in sera of COVID-19 convalescents and non-infected subjects. Differences are statistically significant: *p* = 0.0105 and *p* = 0.0435, respectively.

In our study, we decided to also measure markers of nervous system function. However, both NCAM-1 and S100 protein have another role. NCAM-1, despite being a marker of neurodegeneration, is also expressed on NK (natural killer) cells; thus, its soluble form found in serum may serve as a marker of the number and activity of NK cells. On the other hand, S100 protein might serve as a marker of blood–cerebrospinal fluid barrier permeability and traumatic brain injury, as well as a marker of melanoma. Here, we found that convalescents of COVID-19 have significantly lower S100 and significantly higher NCAM-1 serum concentrations than individuals who have never had contact with SARS-CoV-2 (Figure 3). Exact values of the measurements are summarized in Table 1.

### 3.4. Kidney Markers

**Figure 4:**
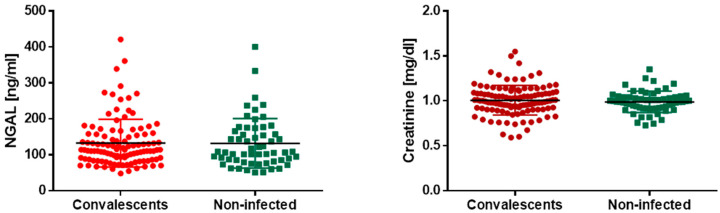
NGAL and creatinine concentrations in sera of COVID-19 convalescents and non-infected subjects. Differences were not statistically significant, *p* > 0.05.

Further, we decided to examine if SARS-CoV-2 infection may lead to recurrent kidney failure. We assessed two widely used kidney function markers—creatinine and NGAL—in the serum of COVID-19 convalescents and subjects never infected with SARS-CoV-2 and found no difference in both markers’ concentration between the study and control group (Figure 4). Exact values of the measurements are summarized in Table 1.

## 4. Discussion

We found that COVID-19 convalescents may present with prolonged dysfunction of the nervous system. However, we did not find a negative impact on the renal system, lung fibrosis, or lung macrophage activation following mild-to-moderate COVID-19 disease at least 6 months prior to the evaluation of healthy individuals.

Acute COVID-19 is associated with respiratory distress and may lead to lung fibrosis. One of the cytokines that play a crucial role in the course of SARS-CoV-2-induced infection is TGF-β, which has been identified as a suppressor of immune response and factors inducing lung fibrosis. Macrophages can produce TGF-β, and their increased activity in the lungs has been confirmed in the course of COVID-19 infection [18]. Several studies have shown that TGF-β mediates extracellular matrix remodeling by activating fibroblasts to proliferate, which leads to edema and fibrosis, resulting in lethal lung injury [5,19]. TGF-β has also been found to be one of the severity markers of COVID-19. Frischbutter et al. showed that patients with severe disease have significantly higher serum concentrations of TGF-β than subjects with moderate or mild COVID-19. They also showed that TGF-β could be used as a predictor of disease fatality, as a higher concentration of this cytokine was found in patients who died than in survivors [20]. A similar positive correlation of TGF-β with the severity of COVID-19 was also found by Susak et al. [21]. Opposite results were shown by a group from Serbia: in their study, deceased patients had significantly lower TGF-β concentrations than survivors. Moreover, they observed differences in TGF-β concentration between COVID-19 subjects dependent on the total platelet (PLT) count in their blood, indicating that the higher the platelet count is, the greater the TGF-β concentration. Interestingly, COVID-19 patients with a normal PLT count did not differ from heathy controls in terms of TGF-β levels [22]. In our study, we were assessing TGF-β in mild-to-moderate COVID-19 convalescents, at least 6 months after recovery. As all included subjects served as blood donors, they did not complain of any symptoms that could be related to lung fibrosis. When comparing convalescents and subjects who have never been infected with SARS-CoV-2, we did not find any difference in TGF-β concentration. Similar results were reported by Sbierski-Kind et al. [23]. We can therefore suggest that either mild-to-moderate healthy COVID-19 convalescents are not at higher risk of the development of pulmonary fibrosis, or that TGF-β is not an appropriate marker of pulmonary fibrosis risk in healthy subjects. This may be determined in a prospective study, in which COVID-19 convalescents would be observed for several years for pulmonary fibrosis development.

As macrophages are the main source of TGF-β, we also assessed markers of monocytes activation in the study group. Soluble CD163 (sCD163) in plasma comes from the surface of activated monocytes/macrophages after enzymatic shedding by ADAM17/TACE metalloproteinase, and its concentration corresponds to the intensity of the inflammatory response [24]. This marker has already been analyzed in the acute phase of COVID-19, with an increased concentration in patients admitted to intensive care units due to severe disease or these with a fatal outcome [24,25,26]. Increases in sCD163 in the course of COVID-19 has also been confirmed in children. Interestingly, in their pediatric study, Mostafa et al. [27] found that the serum concentration of sCD163 remains elevated for at least 3 months after recovery. In our study, we did not find any differences in sCD163 levels between convalescents and never-infected subjects. The study performed by Park et al. showed that convalescents four weeks after recovery still present with an increased number and activity of the monocyte/macrophage population [28]. However, it should also be considered that our study group consisted of healthy individuals who had been infected with SARS-CoV-2 at least 6 months earlier and had fully recovered. Thus, our results are in line with observations made by Rajamanickam et al. [29], who showed decreasing numbers and levels of sCD163 over time in COVID-19 convalescents.

The most confusing result that we obtained was the decreased Lp-PLA2 concentration in the serum of convalescents compared to never-infected subjects. Lp-PLA2 is an enzyme released from macrophages which hydrolyzes oxidized phospholipids on LDL lipoproteins. Its activity leads to the release of several proinflammatory agents that contribute to endothelial damage and atherosclerosis [30]. The majority of published studies indicate that patients infected with SARS-CoV-2, especially those with a severe course of the disease, express an increased concentration of Lp-PLA2 [31,32,33]. Lp-PLA2 has also been considered to be a prognostic marker for the course of COVID-19 [10]. Interestingly, some studies suggest that Lp-PLA2 activity may become a therapeutic target in the course of COVID-19, as its inhibitors have beneficial effects on patients [34]. On the other hand, a Hungarian study did not show any differences in serum Lp-PLA2 between healthy subjects, COVID-19 survivors, and non-survivors [35]. We aimed to find an explanation for the results that we obtained. Firstly, we highlight that both groups included in our study had very low Lp-PLA2 concentrations, as the reference range is less than 200 ng/mL. This is indicative of their health status as subjects with a low risk of cardiovascular events, including atherosclerosis. Still, the significance of a difference is puzzling. We hypothesize that the baseline activity of Lp-PLA2 in included subjects could have been different even before infection with COVID-19, and its lower activity might be a factor contributing to infection due to a delayed inflammatory response. On the other hand, higher Lp-PLA2 activity is associated with increased apolipoprotein B concentration and disrupted lipoproteins balance [36]. Maybe, additional studies on lipid profile assessment in an enrolled group would help to find an answer as to why both groups differed in Lp-PLA2 activity. However, those hypotheses require further studies and solid arguments to be confirmed.

In the present study, two markers were identified as indicative of neurodegeneration (NCAM-1) and brain injury (S100B). NCAM-1, known also as CD56, is also expressed on the surface of natural killer (NK) cells [37]. We found that COVID-19 convalescents had a lower serum concentration of NCAM-1 than those subjects who have never been infected with SARS-CoV-2. In terms of available studies performed on patients with active COVID-19, our result may be misleading. Laudanski et al. [38] reported significantly increased concentrations of this marker in the serum of patients with active COVID-19. Neurodegeneration during the course of COVID-19 may be associated with an immune response against external structures of SARS-CoV-2, which shows molecular mimicry to NCAM-1 found in the central nervous system. Prolonged exposure to immune reactions may lead to Guillain–Barre syndrome [38]. Sun et al. [39] also analyzed the effect of COVID-19 on possible neurodegeneration. They assessed the protein content of neuronal-derived microvesicles in convalescents 1–3 months after active disease and found that the serum of never-infected people contains fewer microvesicles that bear NCAM-1 [38]. As we showed that COVID-19 convalescents are characterized by lower NCAM-1 serum concentrations than never-infected individuals, we suppose that the observed effect may be associated not with neurodegeneration but with possible immune system activity. As previously mentioned, CD56 is a marker of NK cells. Decreases in its NCAM-1/sCD56 concentration may be associated with two phenomena confirmed in the course of infection with SARS-CoV-2. The first one is known as the “exhausted phenotype”. During infection, NK cells may change their phenotype from CD56bright to CD56dim, or even CD56neg. Such cells express a less cytotoxic phenotype, with decreased expression of perforins and granzymes [40,41]. The other mechanism which can lead to decreased CD56 expression is a depletion in the number of NK cells in the peripheral blood of patients suffering from COVID-19 [42]. This is explained by increased NK cell recruitment to the lungs, which was confirmed by Liao et al. [43]. Moreover, it has been shown that in the blood of COVID-19 convalescents 1 to 30 days after recovery, an atypical population of NK cells can be found that express a phenotype characterized by CD56dimCD16neg [44,45]. Unfortunately, we did not have the opportunity to assess NK cell number and phenotype in this study population, which can be identified as a limitation of our study. However, we suppose that changes in immune cell phenotypes may be associated with decreased NCAM-1 in the serum of convalescents.

Our observed increase in S100 protein concentration in the serum of COVID-19 convalescents in our study is consistent with other studies. Aceti et al. and Mete et al. found that S100B concentration is increased in COVID-19 patients, and its concentration rises with disease progression [46,47]. The association between S100B and brain injury in the course of COVID-19 was suggested by Sahin et al., who observed higher S100B concentrations in subjects with at least one neurological symptom [48]. Interestingly, there is an association between gender and S100B concentration, with higher values in males than females [49]. We, however, do not have any information about the sex distribution of our study group, and thus cannot explain the results with this variable. What has to be underlined is that both study groups had a very low S100 concentration, which is not indicative of central nervous system (CNS) pathology. Despite a statistically significant difference in S100 between convalescents and never-infected subjects, we cannot clearly conclude, based on our results, that past COVID-19 infection may influence functioning of the nervous system. However, according to many reports suggesting a negative impact of SARS-CoV-2 infection on the CNS [50], convalescents should pay attention to any neurological symptoms that may appear after the disease.

The last part of our study was to measure renal injury markers in mild-to-moderate COVID-19 convalescents. We found no difference in creatinine or NGAL concentrations between both analyzed groups. However, all included subjects were healthy and usually young, as they qualified as blood donors. However, several studies showed a high utility of renal markers in the acute phase of the disease. Menez et al. [51] found that increased NGAL concentration in the acute phase of the disease was associated with an increased risk of kidney injury in COVID-19 patients. Another group has also shown that NGAL and cystatin C may serve as markers of acute kidney injury (AKI) development in the course of COVID-19, with a distinguishing power similar to creatinine. On the other hand, NGAL was not useful in distinguishing the risk of severe AKI development in subjects infected with SARS-CoV-2 [52]. Our results are in line with results obtained with Serwin et al. They showed that asymptomatic patients with SARS-CoV-2 infection do not present with an increased creatinine concentration compared to healthy subjects. However, they had increased NGAL concentrations, which we did not observe. However, it should be highlighted that they included patients who were sick or had recovered 7, 14, and 28 days earlier, and our study group comprised subjects who had recovered at least 6 months earlier [53]. An interesting study was performed by Malinowska et al. In their study, they analyzed patients following kidney transplantation, who had recovered from COVID-19 6 months earlier. Similar to our results, they did not observe any differences between subjects who had had COVID-19 in the past and subjects who were not infected with SARS-CoV-2, despite all included subjects being kidney transplant recipients [54].

## 5. Conclusions

To conclude, our study shows that mild-to-moderate COVID-19 convalescents are at a low risk of developing lung fibrosis or chronic kidney disease. However, they present some markers of persistent immunological exhaustion at least 6 months after disease recovery. Thus, despite being completely healthy, as they had qualified to become blood donors, they should regularly undergo prophylaxis examinations for the early detection of possible negative outcomes of COVID-19.

## Figures and Tables

**Table 1:** Concentration of all studied markers in convalescents and non-infected subjects. C—convalescents, NI—non-infected, Q1—first quartile, Q3—third quartile.

		Median	Q1	Q3	*p*
TGFβ [pg/mL]	C	159.83	100.75	193.22	*p* = 0.91
	NI	150.66	106.76	208.55	
sCD163 [ng/mL]	C	178.09	143.78	226.73	*p* = 0.42
	NI	175.13	138.45	229.02	
Lp-PLA2 [ng/mL]	C	11.09	4.67	15.26	*p* = 0.028
	NI	12.99	8.6	17.81	
NCAM-1 [pg/mL]	C	2881.78	1875.45	4755.82	*p* = 0.0105
	NI	3728.48	2212.74	6795.65	
S100 [µg/mL]	C	0.040	0.027	0.046	*p* = 0.04
	NI	0.029	0.023	0.044	
NGAL [ng/mL]	C	113.00	87.08	156.25	*p* = 0.77
	NI	107.00	85.13	163.00	
Creatinine [mg/dL]	C	1.00	0.92	1.09	*p* = 0.37
	NI	0.99	0.92	1.04	

## Data Availability

The data presented in this study are available on request from the corresponding author.

## References

[1] Kozłowski P., Leszczyńska A., Ciepiela O. (2024). Long COVID definition, symptoms, risk factors, epidemiology and autoimmunity—A narrative review. Am. J. Med. Open.

[2] Guziejko K., Tałataj J., Czupryna P., Moniuszko-Malinowska A. (2022). Long COVID. Epidemiol. Rev..

[3] Lippi G., Sanchis-Gomar F., Henry B.M. (2023). COVID-19 and its long-term sequelae: What do we know in 2023?. Pol. Arch. Intern. Med..

[4] John A.E., Joseph C., Jenkins G., Tatler A.L. (2021). COVID-19 and pulmonary fibrosis: A potential role for lung epithelial cells and fibroblasts. Immunol. Rev..

[5] Hirawat R., Jain N., Aslam Saifi M., Rachamalla M., Godugu C. (2023). Lung fibrosis: Post-COVID-19 complications and evidences. Int. Immunopharmacol..

[6] Mohammadi A., Balan I., Yadav S., Matos W.F., Kharawala A., Gaddam M., Sarabia N., Koneru S.C., Suddapalli S.K., Marzban S. (2022). Post-COVID-19 Pulmonary Fibrosis. Cureus.

[7] Siddiqi H.K., Libby P., Ridker P.M. (2021). COVID-19—A vascular disease. Trends Cardiovasc. Med..

[8] Vassiliou A.G., Vrettou C.S., Keskinidou C., Dimopoulou I., Kotanidou A., Orfanos S.E. (2023). Endotheliopathy in Acute COVID-19 and Long COVID. Int. J. Mol. Sci..

[9] Tellis C.C., Tselepis A.D. (2014). Pathophysiological role and clinical significance of lipoprotein-associated phospholipase A(2) (Lp-PLA(2)) bound to LDL and HDL. Curr. Pharm. Des..

[10] Dua P., Mishra A., Reeta K.H. (2022). Lp-PLA2 as a biomarker and its possible associations with SARS-CoV-2 infection. Biomark. Med..

[11] Monje M., Iwasaki A. (2022). The neurobiology of long COVID. Neuron.

[12] Leng A., Shah M., Ahmad S.A., Premraj L., Wildi K., Li Bassi G., Pardo C.A., Choi A., Cho S.M. (2023). Pathogenesis Underlying Neurological Manifestations of Long COVID Syndrome and Potential Therapeutics. Cells.

[13] Leone M.A., Helbok R., Bianchi E., Yasuda C.L., Konti M., Ramankulov D., Lolich M., Lovrencic-Huzjan A., Kovacs T., Armon C. (2024). Outcome predictors of post-COVID conditions in the European Academy of Neurology COVID-19 registry. J. Neurol..

[14] Bowe B., Xie Y., Xu E., Al-Aly Z. (2021). Kidney Outcomes in Long COVID. J. Am. Soc. Nephrol..

[15] Rai V. (2023). COVID-19 and Kidney: The Importance of Follow-Up and Long-Term Screening. Life.

[16] Avotins L., Kroica J., Petersons A., Zentina D., Kravale Z., Saulite A., Racenis K. (2023). eGFR(cystatinC)/eGFR(creatinine) ratio < 0.6 in patients with SARS-CoV-2 pneumonia: A prospective cohort study. BMC Nephrol..

[17] Bergersen K.V., Pham K., Li J., Ulrich M.T., Merrill P., He Y., Alaama S., Qiu X., Harahap-Carrillo I.S., Ichii K. (2023). Health disparities in COVID-19: Immune and vascular changes are linked to disease severity and persist in a high-risk population in Riverside County, California. BMC Public Health.

[18] Yao C., Parimon T., Espindola M.S., Hohmann M.S., Konda B., Hogaboam C.M., Stripp B.R., Chen P. (2023). Maladaptive TGF-beta Signals to the Alveolar Epithelium Drive Fibrosis after COVID-19 Infection. Am. J. Respir. Crit. Care Med..

[19] Ramirez-Martinez G., Jimenez-Alvarez L.A., Cruz-Lagunas A., Ignacio-Cortes S., Gomez-Garcia I.A., Rodriguez-Reyna T.S., Choreno-Parra J.A., Zuniga J. (2022). Possible Role of Matrix Metalloproteinases and TGF-beta in COVID-19 Severity and Sequelae. J. Interferon Cytokine Res..

[20] Frischbutter S., Durek P., Witkowski M., Angermair S., Treskatsch S., Maurer M., Radbruch A., Mashreghi M.F. (2023). Serum TGF-beta as a predictive biomarker for severe disease and fatality of COVID-19. Eur. J. Immunol..

[21] Susak F., Vrsaljko N., Vince A., Papic N. (2023). TGF Beta as a Prognostic Biomarker of COVID-19 Severity in Patients with NAFLD-A Prospective Case-Control Study. Microorganisms.

[22] Zivancevic-Simonovic S., Minic R., Cupurdija V., Stanojevic-Pirkovic M., Milosevic-Djordjevic O., Jakovljevic V., Mihaljevic O. (2023). Transforming growth factor beta 1 (TGF-beta1) in COVID-19 patients: Relation to platelets and association with the disease outcome. Mol. Cell. Biochem..

[23] Sbierski-Kind J., Schlickeiser S., Feldmann S., Ober V., Gruner E., Pleimelding C., Gilberg L., Brand I., Weigl N., Ahmed M.I.M. (2024). Persistent immune abnormalities discriminate post-COVID syndrome from convalescence. Infection.

[24] Volfovitch Y., Tsur A.M., Gurevitch M., Novick D., Rabinowitz R., Mandel M., Achiron A., Rubinstein M., Shoenfeld Y., Amital H. (2022). The intercorrelations between blood levels of ferritin, sCD163, and IL-18 in COVID-19 patients and their association to prognosis. Immunol. Res..

[25] Cardelli M., Pierpaoli E., Marchegiani F., Marcheselli F., Piacenza F., Giacconi R., Recchioni R., Casoli T., Stripoli P., Provinciali M. (2022). Biomarkers of cell damage, neutrophil and macrophage activation associated with in-hospital mortality in geriatric COVID-19 patients. Immun. Ageing.

[26] Attia H., El Nagdy M., Halim R.M.A. (2023). Preliminary Study of sCD14 and sCD163 as Predictors of Disease Severity and ICU Admission in COVID-19: Relation to Hematological Parameters, Blood Morphological Changes and Inflammatory Biomarkers. Mediterr. J. Hematol. Infect. Dis..

[27] Mostafa G.A., Ibrahim H.M., Al Sayed Shehab A., Gendy Y.G.E., Aly D.M.M., Shousha G.A.H. (2022). Up-regulated serum levels of soluble CD25 and soluble CD163 in pediatric patients with SARS-CoV-2. Eur. J. Pediatr..

[28] Park J., Dean L.S., Jiyarom B., Gangcuangco L.M., Shah P., Awamura T., Ching L.L., Nerurkar V.R., Chow D.C., Igno F. (2023). Elevated circulating monocytes and monocyte activation in COVID-19 convalescent individuals. Front. Immunol..

[29] Rajamanickam A., Kumar N.P., Pandiarajan A.N., Selvaraj N., Munisankar S., Renji R.M., Venkatramani V., Murhekar M., Thangaraj J.W.V., Kumar M.S. (2021). Dynamic alterations in monocyte numbers, subset frequencies and activation markers in acute and convalescent COVID-19 individuals. Sci. Rep..

[30] Xu X.Y., Guo L., Wang Q., Yu X.B., Li L., Wei Q. (2020). Association between lipoprotein-associated phospholipase A(2) and lower extremity arterial disease in type 2 diabetes mellitus. Clin. Chim. Acta.

[31] Li Y., Jiang Y., Zhang Y., Li N., Yin Q., Liu L., Lv X., Liu Y., Li A., Fang B. (2021). Abnormal upregulation of cardiovascular disease biomarker PLA2G7 induced by proinflammatory macrophages in COVID-19 patients. Sci. Rep..

[32] Carmo H.R.P., Yoshinaga M.Y., Castillo A.R., Britto Chaves-Filho A., Bonilha I., Barreto J., Muraro S.P., de Souza G.F., Davanzo G.G., Perroud M.W. (2023). Phenotypic changes in low-density lipoprotein particles as markers of adverse clinical outcomes in COVID-19. Mol. Genet. Metab..

[33] Gravrand V., Mellot F., Ackermann F., Ballester M.C., Zuber B., Kirk J.T., Navalkar K., Yager T.D., Petit F., Pascreau T. (2023). Stratification of COVID-19 Severity Using SeptiCyte RAPID, a Novel Host Immune Response Test. Viruses.

[34] Batsika C.S., Gerogiannopoulou A.D., Mantzourani C., Vasilakaki S., Kokotos G. (2021). The design and discovery of phospholipase A(2) inhibitors for the treatment of inflammatory diseases. Expert Opin. Drug Discov..

[35] Suto R., Pocsi M., Fagyas M., Kalina E., Fejes Z., Szentkereszty Z., Kappelmayer J., Nagy B. (2024). Comparison of Different Vascular Biomarkers for Predicting In-Hospital Mortality in Severe SARS-CoV-2 Infection. Microorganisms.

[36] Ling Y., Tang S., Cao Y., Fu C. (2020). Relationship between Plasma Lipoprotein-Associated Phospholipase A2 Concentrations and Apolipoprotein in Stable Coronary Artery Disease Patients. Dis. Markers.

[37] Van Acker H.H., Capsomidis A., Smits E.L., Van Tendeloo V.F. (2017). CD56 in the Immune System: More Than a Marker for Cytotoxicity?. Front. Immunol..

[38] Laudanski K., Hajj J., Restrepo M., Siddiq K., Okeke T., Rader D.J. (2021). Dynamic Changes in Central and Peripheral Neuro-Injury vs. Neuroprotective Serum Markers in COVID-19 Are Modulated by Different Types of Anti-Viral Treatments but Do Not Affect the Incidence of Late and Early Strokes. Biomedicines.

[39] Sun B., Tang N., Peluso M.J., Iyer N.S., Torres L., Donatelli J.L., Munter S.E., Nixon C.C., Rutishauser R.L., Rodriguez-Barraquer I. (2021). Characterization and Biomarker Analyses of Post-COVID-19 Complications and Neurological Manifestations. Cells.

[40] Björkström N.K., Strunz B., Ljunggren H.G. (2022). Natural killer cells in antiviral immunity. Nat. Rev. Immunol..

[41] Saresella M., Trabattoni D., Marventano I., Piancone F., La Rosa F., Caronni A., Lax A., Bianchi L., Banfi P., Navarro J. (2021). NK Cell Subpopulations and Receptor Expression in Recovering SARS-CoV-2 Infection. Mol. Neurobiol..

[42] Maucourant C., Filipovic I., Ponzetta A., Aleman S., Cornillet M., Hertwig L., Strunz B., Lentini A., Reinius B., Brownlie D. (2020). Natural killer cell immunotypes related to COVID-19 disease severity. Sci. Immunol..

[43] Liao M., Liu Y., Yuan J., Wen Y., Xu G., Zhao J., Cheng L., Li J., Wang X., Wang F. (2020). Single-cell landscape of bronchoalveolar immune cells in patients with COVID-19. Nat. Med..

[44] Leem G., Cheon S., Lee H., Choi S.J., Jeong S., Kim E.S., Jeong H.W., Jeong H., Park S.H., Kim Y.S. (2021). Abnormality in the NK-cell population is prolonged in severe COVID-19 patients. J. Allergy Clin. Immunol..

[45] Casado J.L., Moraga E., Vizcarra P., Velasco H., Martin-Hondarza A., Haemmerle J., Gomez S., Quereda C., Vallejo A. (2021). Expansion of CD56(dim)CD16(neg) NK Cell Subset and Increased Inhibitory KIRs in Hospitalized COVID-19 Patients. Viruses.

[46] Aceti A., Margarucci L.M., Scaramucci E., Orsini M., Salerno G., Di Sante G., Gianfranceschi G., Di Liddo R., Valeriani F., Ria F. (2020). Serum S100B protein as a marker of severity in COVID-19 patients. Sci. Rep..

[47] Mete E., Sabirli R., Goren T., Turkcuer I., Kurt O., Koseler A. (2021). Association Between S100b Levels and COVID-19 Pneumonia: A Case Control Study. In Vivo.

[48] Sahin B.E., Celikbilek A., Kocak Y., Saltoglu G.T., Konar N.M., Hizmali L. (2022). Plasma biomarkers of brain injury in COVID-19 patients with neurological symptoms. J. Neurol. Sci..

[49] Savarraj J., Park E.S., Colpo G.D., Hinds S.N., Morales D., Ahnstedt H., Paz A.S., Assing A., Liu F., Juneja S. (2021). Brain injury, endothelial injury and inflammatory markers are elevated and express sex-specific alterations after COVID-19. J. Neuroinflamm..

[50] Tang N., Kido T., Shi J., McCafferty E., Ford J.M., Dal Bon K., Pulliam L. (2024). Blood Markers Show Neural Consequences of LongCOVID-19. Cells.

[51] Menez S., Moledina D.G., Thiessen-Philbrook H., Wilson F.P., Obeid W., Simonov M., Yamamoto Y., Corona-Villalobos C.P., Chang C., Garibaldi B.T. (2022). Prognostic Significance of Urinary Biomarkers in Patients Hospitalized With COVID-19. Am. J. Kidney Dis..

[52] Pode Shakked N., de Oliveira M.H.S., Cheruiyot I., Benoit J.L., Plebani M., Lippi G., Benoit S.W., Henry B.M. (2022). Early prediction of COVID-19-associated acute kidney injury: Are serum NGAL and serum Cystatin C levels better than serum creatinine?. Clin. Biochem..

[53] Serwin N., Cecerska-Heryc E., Pius-Sadowska E., Serwin K., Niedzwiedz A., Wisniewska M., Roszak M., Grygorcewicz B., Skwirczynska E., Machalinski B. (2022). Renal and Inflammation Markers-Renalase, Cystatin C, and NGAL Levels in Asymptomatic and Symptomatic SARS-CoV-2 Infection in a One-Month Follow-Up Study. Diagnostics.

[54] Malinowska A., Heleniak Z., Muchlado M., Slizien Z., Ruszkowski J., Biedunkiewicz B., Tylicki L., Krol E., Debska-Slizien A. (2022). Changes in Kidney Graft Function in COVID-19 Convalescents. Transplant. Proc..

